# Monitoring the process mean under the Bayesian approach with application to hard bake process

**DOI:** 10.1038/s41598-023-48206-1

**Published:** 2023-11-25

**Authors:** Imad Khan, Muhammad Noor-ul-Amin, Dost Muhammad Khan, Emad A. A. Ismail, Uzma Yasmeen, Javed Rahimi

**Affiliations:** 1 Department of Statistics, Abdul Wali Khan University Mardan, Mardan, Pakistan; 2 Department of Statistics, COMSATS University Islamabad, Lahore Campus, Lahore, Pakistan; 3 Department of Quantitative Analysis, College of Business Administration, King Saud University, P.O. Box 71115, Riyadh, 11587 Saudi Arabia; 4 Department of Mathematics and Statistics, BROCK University, St Catharines, Canada; 5 Kabul City Agriculture and Food Processing Institute, Kabul, Afghanistan

**Keywords:** Engineering, Computational science, Statistics

## Abstract

This study introduces the Bayesian adaptive exponentially weighted moving average (AEWMA) control chart within the framework of measurement error, examining two separate loss functions: the squared error loss function and the linex loss function. We conduct an analysis of the posterior and posterior predictive distributions utilizing a conjugate prior. In the presence of measurement error (ME), we employ a linear covariate model to assess the control chart's effectiveness. Additionally, we explore the impacts of measurement error by investigating multiple measurements and a method involving linearly increasing variance. We conduct a Monte Carlo simulation study to assess the control chart's performance under ME, examining its run length profile. Subsequently, we offer a specific numerical instance related to the hard-bake process in semiconductor manufacturing, serving to verify the functionality and practical application of the suggested Bayesian AEWMA control chart when confronted with ME.

## Introduction

Statistical Process Control (SPC) is a powerful and widely used quality management approach that empowers organizations to achieve process improvements, minimize variability, and ensure the consistent delivery of high-quality products and services. By leveraging data-driven decision-making and driving continuous process improvements, SPC makes a vital contribution to maintaining customer satisfaction and achieving competitiveness in dynamic markets. Originated by Walter A. Shewhart and W. Edwards Deming, the fundamental principle of SPC is the ability to distinguish between common and special cause variations within processes and enable companies to locate and remediate the sources of special cause variations to optimize process control. Using statistical tools such as control charts and root cause analysis, SPC provides valuable insights for taking corrective and preventive actions, resulting in significant reductions in defects, waste, and operational costs, further enhancing a company's overall efficiency and profitability. To conclude, SPC and control charts (CCs)are essential for maintaining process stability, and product quality, and facilitating ongoing enhancements., enabling organizations to stay competitive and meet the demands of a dynamic market environment. Shewhart^1^ introduced CCs that operate without the use of historical data, relying exclusively on present sample information to effectively detect significant changes in production processes. In contrast, memory-type CCs, such as the cumulative sum (CUSUM) and exponentially weighted moving average (EWMA) CCs suggested by^2^ and^3^, integrated both current and historical sample data into their computations. Notably, the CUSUM and EWMA CCs exhibit a heightened sensitivity to identifying minor to moderate shifts in process parameters, surpassing the capabilities of the traditional Shewhart CC. These memory-type CCs, especially the EWMA and CUSUM variants, are widely applied in the fields of chemical and industrial production processes. Herdiani et al.^4^ studied CCs in SPC are adapted to address the violation of mutually independent observations, especially for interrelated processes with autocorrelation, by using time series models and the Markov Chain Method to evaluate the ARL performance of the mean of EWMA in autoregressive processes. Wu et al.^5^ introduced a distribution-free upper-sided EWMA CC to monitor both time intervals and magnitudes of events without relying on known distributions. The method utilizes 'continuousify' and classical Markov chain analysis to ensure reliable run length properties. Numerical comparison with the parametric Shewhart TBEA chart confirms the effectiveness of this approach, demonstrated through an example using a French forest fire database. Alevizakos et al.^6^ presented the Triple EWMA (TEWMA) CC, outperforming other charts in detecting small and moderate shifts in process mean. Monte Carlo simulations confirm its effectiveness, showing superior performance for small shifts and comparable results for moderate and large shifts, with better inertia properties and robustness for small smoothing parameters. CCs serve as a beneficial tool when shift size information is available or when analyzing specific shifts. Nevertheless, in certain instances, the shift size may be predetermined prior to using CCs. Quality investigators can enhance the detection capability for changes of varying magnitudes by exploring double and adaptive control charting techniques. Ali et al.^7^ investigated the NPMEPSN chart, utilizing both simple and RSS methods to effectively detect small shifts, and demonstrated its robust performance across various distributions. The enhanced version (NPMEPRSN) under the RSS scheme exhibited superior performance compared to alternatives, confirmed through simulations and a real dataset concerning piston ring diameter. Noor-ul-Amin and Sarwar^8^ presented a function-based AEWMA CC for monitoring the variance–covariance matrix in a multivariate normal process with composite observations, demonstrating superior performance in covariance matrix detection compared to existing EWMA and AEWMA charts. An example using data from the bimetal thermostat industry highlights the chart's practical effectiveness. Noor-ul-Amin and Sarwar^9^ proposed an AMEWMA CC for detecting mean vector variations in a multivariate normal distribution, demonstrating superior shift detection compared to MEWMA and existing AMEWMA charts through Monte Carlo simulations. A real-life case study on process capability for turning aluminum pins exemplifies the practical application of the proposed chart, monitoring six quality characteristics. Zaman et al.^10^ proposed an AEWMA using Huber and Tukey's bisquare functions to monitor shifts of varying sizes in manufacturing and non-manufacturing processes. Performance measures, like ARL and extra quadratic loss, show its competitive efficiency in detecting small to large shifts, validated through an illustrative example with real data. CCs have enhanced capabilities in detecting minor to moderate disturbances, but their efficiency is affected when measurement errors (ME) occur during data collection for constructing the charts. ME can cause variations in the measured study variables, leading to quality disruptions and undesirable results, consequently diminishing the ability of CCs to detect out-of-control signals. Researchers have introduced various methods to address this challenge of ME in CCs. Maravelakis et al.^11^ investigated the influence of ME on the EWMA CCs capability to detect out-of-control situations, particularly focusing on a shift in mean with linear covariates. The study also explores the impact of multiple measurements on each sampled unit and linearly increasing variance, concluding that ME significantly affects the chart's performance in detecting mean shifts. FAEWMA-ME-CV CC, considering ME, linear covariate model, and multiple measurements, efficiently detects infrequent process changes in the form of parametric shifts in the process coefficient of variation (CV) was studied by Arshad et al.^12^. Tang et al.^13^ investigated the impact of measurement errors on the AEWMA median chart's efficiency, proposing a parameter optimization strategy, and demonstrate its superiority over Shewhart and classical EWMA schemes, emphasizing the importance of multiple measurements per sample point. Yang et al.^14^ proposed an error-corrected dispersion CC, using a corrected sign statistic and an EWMA, effectively addressing MEs and improving control limits for process dispersion monitoring. Numerical analyses demonstrate the chart's capability to handle substantial measurement error levels, validated by its successful application in semiconductor data. Zaidi et al.^15^ worked on monitoring compositional data using the MEWMA-CoDa chart, exploring the impact of MEs and showcasing its superior performance in detecting shifts. The study assesses device parameters (σM, b) influence alongside independent observations (m) and variables (p), exemplified through a muesli production case. Evidence from earlier studies shows a prevalent use of the conventional methodology, which relies solely on sample data without incorporating prior knowledge. In contrast, the Bayesian approach combines sample data with prior knowledge to update and generate a posterior (P) distribution, thereby enhancing the estimation process. A Bayesian CC utilizing the P distribution to monitor the process mean was introduced by Saghir et al.^16^. Their methodology incorporates various LFs, providing adaptability to capture inherent process characteristics. Noor-ul-Amin and Noor^17^ studied novel AEWMA CC is proposed for process mean monitoring using Bayesian theory, informed by different LFs, evaluated via ARL and SDRL, compared to existing Bayesian EWMA, and validated with Monte Carlo simulations and a real-data case. Jones et al.^18^ adapted CUSUM and EWMA CCs to a Bayesian framework, utilizing P distributions and different LFs. Applied to count data, simulations evaluate performance by considering shift size sensitivity and hyper-parameters, along with a real data application. Noor-ul-Amin and Noor^19^ introduced a Bayesian chart with SELF and LLF, addressing ME through P and posterior predictive (PP) distributions, evaluated via a linear covariate model, multiple measurements, and linearly increasing variance methods, and validated using run length profiles, Monte Carlo simulations, and a real-life data example. Khan et al.^20^ explored the performance of Bayesian-AEWMA control charts using RSS and two various LFs, incorporating MEs, a covariate model, and multiple measurement techniques. Assessment through run length profiles, alongside a semiconductor application, emphasizes its efficiency in identifying out-of-control signals, favoring the MRSS approach for managing MEs.

All the aforementioned work has been carried out for both the classical and Bayesian approaches. The main motivation of the current study is to address the issue of measurement error through CC using Bayesian methodology. We introduce a Bayesian AEWMA CC that incorporates ME using conjugate priors and shows its performance in the presence of ME. It accommodates two various LF i.e., (SELF) and false negatives (LLF) and employs three ME methods: (i) covariate, (ii) multiple measurements, and (iii) linearly increasing variance. Performance assessment utilizes ARL and SDRL, determined through Monte Carlo simulations. The structure of this paper is as follows: In Section “Bayesian approach”, we delve into the Bayesian methodology applied to the AEWMA control chart and discuss the utilized LFs. Section “Measurement error” is dedicated to exploring ME, while in Section “Suggested Bayesian AEWMA CC using lf under me”, we introduce the suggested implementation of the AEWMA CC with ME through Bayesian techniques. Discussions and key findings are summarized in Section “Discussion on tables and main findings”, and real-life data applications are showcased in Section “Real life data applications”. Section “Conclusion” concludes the article, while Section “Limitations of the study and future recommendations” addresses the limitations and provides recommendations.

## Bayesian approach

The Bayesian methodology provides an alternative perspective compared to the frequentist (classical) approach, treating the parameter as a “random variable” following a prior distribution defined by hyperparameters. The P distribution is constructed using two categories of prior distributions: non-informative and informative. Non-informative priors, like Jeffreys and uniform priors, are frequently employed, while informative priors often rely on conjugate priors as a prominent family. Let’s examine the study variable *X* in an in-control process defined by parameters $\theta$ (mean) and $\delta^{2}$ (variance). In this context, a normal prior is utilized, with $\theta_{0}$ and $\delta_{0}^{2}$ serving as its corresponding parameters and defined as:

1 $$p\left( \theta \right) = \frac{1}{{\sqrt {2\pi \delta_{0}^{2} } }}\exp \left\{ { - \frac{1}{{2\delta_{0}^{2} }}\left( {\theta - \theta_{0} } \right)^{2} } \right\}$$

Formulating the P distribution involves combining the likelihood function of the sampling distribution and the prior distribution, resulting in a proportional relationship achieved through multiplication. Consequently, the resulting P distribution representing the unknown parameter $\theta$, based on the observed data *x*, can be expressed as:

2 $$p\left( {\theta |x} \right) = \frac{{p\left( {x|\theta } \right)p\left( \theta \right)}}{{\int {p\left( {x|\theta } \right)p\left( \theta \right)d\theta } }}$$

The PP distribution is utilized to forecast future observations using the prior distribution while integrating data-derived information. Often used as a prior distribution for new data Y, it enables predictions for upcoming observations with consideration of uncertainty. Integral to Bayesian theory, the PP distribution allows for updating prior distributions with new data. Its mathematical representation is provided below:

3 $$p\left( {y|x} \right) = \int {p\left( {y|\theta } \right)p\left( {\theta |x} \right)d\theta }$$

### Loss functions

In the Bayesian approach, the “loss function” is essential for informed decision-making, quantifying costs tied to choices and aiding trade-offs. It bridges statistical analysis and decision-making, guiding choices under uncertainty, and integrating it enhances the Bayesian framework's holistic decision-making, optimizing options and refining models across fields like quality control and parameter estimation. In the current study we considered both the symmetric and asymmetric LFs. The Squared Error loss function (SELF) is commonly utilized as a symmetric LF in the context of Bayesian inference, as suggested by Gauss^21^. If the predictive variable *X* and $\hat{\theta }_{(SELF)}$ is its estimate then SELF is mathematically described as:

4 $$L\left( {X,\hat{\theta }_{(SELF)} } \right) = \left( {X - \hat{\theta }_{(SELF)} } \right)^{2}$$

and the Bayes estimator, which minimized the SELF is given below:

5 $$\hat{\theta }_{(SELF)} = E_{\theta /x} \left( \theta \right).$$

Varian^22^ introduced the LINEX loss function (LLF), an asymmetric LF tailored to scenarios where the impact of overestimation is pronounced. LLF allows a versatile assessment of the balance between underestimation and overestimation, making it valuable when one type of error holds greater importance than the other, especially in situations where the cost or consequences of overestimating a parameter or outcome are of greater concern than underestimation. The LLF is mathematically presented as:

6 $$L\left( {\theta ,\hat{\theta }_{(LLF)} } \right) = \exp \left( {c\left( {\theta - \hat{\theta }_{(LLF)} } \right)} \right) - c\left( {\theta - \hat{\theta }_{(LLF)} } \right) - 1$$

Using LLF, Bayes estimator is described as:

7 $$\hat{\theta }_{(LLF)} = - \frac{1}{c}InE_{\theta /x} \left( {e^{ - c\theta } } \right).$$

## Measurement error

Measurement error, arising from factors like instrument precision, human mistakes, and environmental variability, can significantly affect data accuracy across diverse fields. This phenomenon introduces discrepancies between actual values and recorded measurements, posing challenges in research by distorting relationships, statistical inferences, and conclusions. Recognizing its importance is vital for researchers and practitioners, as understanding and addressing ME impacts data validity, decision-making, and research quality, underscoring the need for robust strategies to enhance data integrity and interpretation. This study utilizes the covariate model to manage ME, employing the strategy of multiple measurements to minimize its impact. This process entails gathering multiple measurements for each observation, resulting in enhanced precision when estimating the actual underlying value. Additionally, the linearly increasing variance method is also discussed to address the issue of ME.

### Covariate model

Bennett^23^ offered a model for assessing the influence of ME on the Shewhart control chart, described as Y = X + e. In this equation, X represents the study variable, following a normal distribution with a mean of and a variance of $\delta^{2}$. This framework pertains to the in-control process, where $\varepsilon$ captures stochastic error arising from measurement imprecision. Linna and Woodall^24^ subsequently investigated the covariate model, outlined as:

8 $$Y = A + BX + \varepsilon$$

The model incorporates constants A and B, alongside a normally distributed variable with a mean of zero and variance $\delta_{m}^{2}$. All parameters in the model are presumed to be known, and X and e are treated as independent variables. i.e., $Cov\left( {X,\varepsilon } \right) = 0$, and the variable under consideration *Y* is also distributed normally having mean $A + B\theta$ and variance $B^{2} \delta^{2} + \delta_{m}^{2}$.

### Multiple measurements method

Walden^25^ adopted a strategy of employing multiple measurements for each sampling unit, substituting a single measurement, thereby mitigating the variation induced by ME. As the number of multiple measurements increases indefinitely, the variance of the ME component approaches zero. Notably, implementing multiple measurements without ME does not affect the performance of CCs techniques. When employing multiple measurements and considering a sample size of m, the variance of the overall mean can be formulated as:

9 $$\left( {\frac{{B^{2} \delta^{2} }}{n} + \frac{{\delta_{m}^{2} }}{nk}} \right)$$

### Linearly increasing variance method

Within Section “Measurement error”, a covariate model was explored, assuming a consistent variance. Let us utilize the identical model as *Y* = A + B*X* + e, wherein the variance alters linearly in response to fluctuations in the variable *Y*. Here, the term e adheres to a normal distribution with an average of 0 and a variance of *C* + *D*$\theta$. Subsequently, *Y* follows a normal distribution with an average of *A* + *B*$\theta$ and a variance of $B^{2} \delta^{2} + C + D\theta$.

## Suggested Bayesian AEWMA CC using lf under me

The recommended Bayesian CC considering ME to identify unusual fluctuations in the location parameter of the normally distributed process mean. Let X_1_, X_2_, …, Xn denote a sequence of independent and identically distributed random variables, each adhering to a normal distribution with a mean of θ and a variance of δ^2^. The corresponding probability density function is expressed as follows:

10 $$f\left( {x_{t} :\theta ,\sigma^{2} } \right) = \frac{1}{{\sqrt {2\pi \delta^{2} } }}\exp \left( { - \tfrac{1}{{2\delta^{2} }}\left( {x_{t} - \theta } \right)^{2} } \right)$$

Consider the computed mean shift estimate ${\widehat{\delta }}_{t}^{*}$ as an AEWMA sequence originating from {*X*_t_}, illustrated as:

11 $$\widehat{{\delta_{t}^{*} }} = \psi X_{t} + \left( {1 - \psi } \right)\widehat{{\delta_{t - 1}^{*} }}$$

In this scenario, with $\widehat{{\delta_{0}^{*} }}$ set to 0 and *ψ* as the smoothing constant, the estimator $\widehat{{\delta_{t}^{*} }}$ shows an unbiased in the in-control scenario, yet exhibits bias in an out-of-control process. To achieve unbiased measurement in both cases, Haq et al. ^7^ proposed the adoption of $\widehat{{\delta_{t}^{*} }}$, defined as follows:

12 $$\widehat{{\delta_{t}^{**} }} = \frac{{\widehat{{\delta_{t}^{*} }}}}{{1 - \left( {1 - \psi } \right)^{t} }}$$

The author recommended to utilize $$\widehat{{\delta_{t} }} = \left| {\widehat{{\delta_{t}^{**} }}} \right|$$ for estimating $$\delta$$.

The offered AEWMA CC applying Bayesian approach for detecting the process mean utilizing the sequence of $\{ X_{t} \}$ is mathematically described as

13 $$Z_{t} = g\left( {\hat{\delta }_{t} } \right)\hat{\theta }_{(SELF)} + \left( {1 - g\left( {\hat{\delta }_{t} } \right)} \right)Z_{t - 1}$$

where $v\left( {\hat{\delta }_{t} } \right) \in \left( {0,\left. 1 \right]} \right.$ and $Z_{0} = 0$ such that

14 $$v\left( {\hat{\delta }_{t} } \right) = \left\{ {\begin{array}{*{20}l} {\frac{1}{{a\left[ {1 + \left( {\hat{\delta }_{t} } \right)^{ - c} } \right]}}} \hfill & {if} \hfill & {0 < \hat{\delta }_{t} \le 2.7} \hfill \\ 1 \hfill & {if} \hfill & {\hat{\delta }_{t} > 2.7} \hfill \\ \end{array} } \right.$$

Atif et al.^26^ proposed the application of the function described in equation (24) to dynamically modify the smoothing constant value, responding to the estimated shift. The suggested values for the constants used in the function $v(\hat{\delta }_{t} )$ are *a* = 7 and *c* = 1 when the estimated shift $\hat{\delta }_{t}$ falls within the range of 1 to 2.7. Additionally, the constant *c* = 2 for estimated shift values $\hat{\delta }_{t}$ that are less than or equal to 1. Establishing whether the process is within control or beyond control depends on whether the plotting statistic of the Bayesian AEWMA surpasses a predetermined threshold value identified as *h*. Alternatively, the process is considered to be under control if the plotting statistic remains below this threshold.

If both the likelihood function and the prior distribution conform to normal distributions, the resultant P distribution takes on a Gaussian form, characterized by a mean denoted as *θn* and a variance represented by $\delta_{n}^{2}$. This culminates in the establishment of a probability density function, which can be expressed as follows:

15 $$P\left( {\theta |y} \right) = \frac{1}{{\sqrt {2\pi } \sqrt {\frac{{\delta^{2} \delta_{0}^{2} }}{{\delta^{2} + n\delta_{0}^{2} }}} }}\exp \left[ { - \frac{1}{2}\left( {\frac{{\theta - \sum\limits_{i = 1}^{n} {\frac{{y_{i} \delta_{0}^{2} + \theta_{0} \delta_{0}^{2} }}{{\delta^{2} + n\delta_{0}^{2} }}} }}{{\sqrt {\frac{{\delta^{2} \delta_{0}^{2} }}{{\delta^{2} + n\delta_{0}^{2} }}} }}} \right)^{2} } \right]$$

where $$\theta /Y \sim N\left( {\theta_{n} ,\delta_{n}^{2} } \right)$$, *i.e.,* $$\theta_{n} = \frac{{n\overline{y}\delta_{0}^{2} + \delta^{2} \theta_{0} }}{{\delta^{2} + n\delta_{0}^{2} }}$$ and $$\delta_{n}^{2} = \frac{{\delta^{2} \delta_{0}^{2} }}{{\delta^{2} + n\delta_{0}^{2} }}$$.

### Suggested Bayesian CC under ME utilizing SELF using P and PP with covariate model

The Bayes estimator utilized in the proposed Bayesian AEWMA CC under SELF with covariate model is given by:

16 $$\hat{\theta }_{psc(SELF)} = \frac{{n\overline{y}\delta_{0}^{2} + \left( {B^{2} \delta^{2} + \delta_{m}^{2} } \right)\theta_{0} }}{{n\delta_{0}^{2} + B^{2} \delta^{2} + \delta_{m}^{2} }}$$

### Recommended Bayesian CC under ME under SELF using P and PP for multiple measurement method

The Bayes estimator based on the suggested CC using SELF for P and PP distribution under multiple measurements method is mathematized as:

17 $$\hat{\theta }_{psmm(SELF)} = \frac{{n\overline{y}\delta_{0}^{2} + \left( {\frac{{B^{2} \delta^{2} }}{n} + \frac{{\delta_{m}^{2} }}{nk}} \right)\theta_{0} }}{{n\delta_{0}^{2} + \left( {\frac{{B^{2} \delta^{2} }}{n} + \frac{{\delta_{m}^{2} }}{nk}} \right)}}.$$

### Proposed Bayesian AEWMA CC under ME under SELF using P and PP for linearly increasing variance method

The estimator under SELF using suggested AEWMA utilizing Bayesian approach based on the linearly increasing variance method is described as

18 $$\hat{\theta }_{psl(SELF)} = \frac{{n\overline{y}\delta_{0}^{2} + \left( {B^{2} \delta^{2} + C + D\theta } \right)\theta_{0} }}{{n\delta_{0}^{2} + \left( {B^{2} \delta^{2} + C + D\theta } \right)}}.$$

Appendix A contains the estimators for the proposed Bayesian AEWMA CC, which incorporates an informative prior for both the covariate model and the multiple measurements method, along with the linearly increasing variance approach under LLF. Furthermore, Appendix A contains the comprehensive R codes necessary for assessing the run length profile, thus enabling the effective use and execution of the suggested Bayesian AEWMA CC.

## Discussion on tables and main findings

Tables 1, 2 and 3 serve as a platform to showcase the outcomes obtained through the application of the Bayesian AEWMA CC approach, both in scenarios with and without ME considerations. The analysis at hand meticulously considers the influence of two distinct LFs, which are tailored to emphasize the P and PP distributions. These evaluations are conducted within the context of informative priors, strategically employed through the covariate method. Tables 4 and 5 likely present the steady-state ARL that highlights the effectiveness of the suggested CC, emphasizing its proficiency in recognizing and addressing ME, ultimately demonstrating its superiority over other methods in the same context. In a parallel manner, Tables 7, 8, 9 and Tables 10, 11, 12 adhere to the same structured approach, yet they encompass a more comprehensive perspective by accommodating multiple measurements originating from the same sampled values. This expanded scope is complemented by the utilization of the linearly increasing variance method, adding another layer of sophistication to the analysis.

**Table 1:** ARL and SDRL outcomes for CC in the context of ME, within the SELF framework for the covariate model, with $\lambda$ = 0.10.

Shift	No error		0.1		0.2		0.5		1	
	ARL	SDRL	ARL	SDRL	ARL	SDRL	ARL	SDRL	ARL	SDRL
0.0	369.89	422.56	370.68	424.73	371.01	428.22	371.29	433.18	372.81	432.19
0.10	163.12	162.36	172.06	173.93	178.53	179.22	196.80	201.95	218.86	230.10
0.20	75.86	70.03	81.42	75.09	85.23	79.20	97.19	90.5	116.43	110.95
0.30	43.86	39.78	46.95	42.94	49.91	45.06	58.50	53.20	71.93	65.61
0.40	28.00	25.87	29.88	27.64	32.63	29.66	38.35	34.81	47.11	43.00
0.50	18.99	17.61	20.67	19.14	22.29	20.66	26.90	24.60	33.86	31.12
0.60	13.75	12.64	15.00	13.80	16.13	14.87	19.58	18.14	25.38	23.50
0.70	10.41	9.53	11.32	10.29	12.19	11.20	15.08	13.94	19.68	18.04
0.80	8.05	7.15	8.81	7.87	9.55	8.62	11.76	10.79	15.32	14.17
0.90	6.53	5.73	7.03	6.20	7.69	6.81	9.38	8.58	12.48	11.46
1.0	5.37	4.54	5.91	5.08	6.28	5.48	7.77	6.98	10.10	9.25
1.5	2.69	1.92	2.90	2.12	3.13	2.33	3.74	2.93	4.82	4.07
2.0	1.76	1.03	1.88	1.14	1.98	1.24	3.74	2.94	2.94	2.20
2.5	1.36	0.63	1.43	0.71	1.50	0.78	1.71	0.98	2.07	1.33
3	1.16	0.40	1.20	0.45	1.23	0.49	1.38	0.65	1.63	0.91

**Table 2:** Using covariate model, run-length results Bayesian chart in the presence of ME under LLF, with $\lambda$ = 0.10.

Shift	No error		0.1		0.2		0.5		1	
	ARL	SDRL	ARL	SDRL	ARL	SDRL	ARL	SDRL	ARL	SDRL
0.0	369.89	422.56	371.82	421.73	373.51	430.20	372.37	416.16	371.56	431.71
0.10	163.59	161.87	171.87	173.02	183.34	180.85	194.25	200.30	216.14	229.18
0.20	76.32	69.06	81.81	75.10	86.01	78.83	96.49	90.53	115.43	108.91
0.30	43.86	39.78	46.88	42.94	50.13	45.32	57.91	53.31	70.77	65.11
0.40	27.94	25.49	30.44	27.88	32.48	29.57	37.69	34.78	47.81	43.64
0.50	19.17	17.76	21.04	19.55	22.36	20.69	26.75	24.69	33.76	31.48
0.60	13.73	12.67	14.92	13.83	16.28	15.14	19.51	18.15	25.39	23.49
0.70	10.41	9.45	11.35	10.46	12.15	11.26	14.88	13.99	19.57	18.03
0.80	8.07	7.20	8.86	7.98	9.65	8.78	11.72	10.76	15.18	14.37
0.90	6.52	5.71	7.14	6.29	7.73	6.89	9.30	8.46	12.35	11.51
1.0	5.36	4.55	5.89	2.89	6.35	5.55	7.74	6.85	10.21	9.24
1.5	2.69	1.92	2.89	2.10	3.13	2.35	3.71	2.93	4.73	3.98
2.0	1.76	1.04	1.87	1.14	1.99	1.2	2.34	1.56	2.92	2.16
2.5	1.36	0.62	1.43	0.70	1.50	0.77	1.70	0.98	2.07	1.33
3	1.16	0.40	1.20	0.45	1.24	0.50	1.38	0.65	1.62	0.90

**Table 3:** The run length profiles for AEWMA CC under ME with LLF for covariate model, with $\lambda$ = 0.10.

Shift	No error		0.1		0.2		0.5		1	
	ARL	SDRL	ARL	SDRL	ARL	SDRL	ARL	SDRL	ARL	SDRL
0.0	369.29	421.99	371.778	431.56	370.32	423.50	372.71	433.04	373.40	427.34
0.10	164.38	165.33	171.81	171.25	179.45	181.07	198.40	203.87	216.24	227.67
0.20	75.69	69.56	81.84	75.07	85.25	79.65	98.06	91.72	114.56	108.85
0.30	43.99	39.93	47.30	42.83	32.34	29.68	58.18	52.97	70.57	64.57
0.40	27.86	25.71	30.36	27.81	22.55	20.97	38.56	35.06	48.10	43.70
0.50	19.27	17.80	20.85	19.34	16.20	14.96	26.95	24.78	33.90	31.28
0.60	13.71	12.74	15.00	13.91	12.37	11.28	19.91	18.51	25.09	23.36
0.70	10.46	9.59	11.32	10.39	9.69	8.79	11.77	10.88	19.36	18.08
0.80	8.13	7.33	8.85	8.05	7.61	6.72	9.59	8.62	15.16	14.16
0.90	6.60	5.80	7.17	6.31	6.33	5.51	7.74	6.98	12.04	11.25
1.0	5.42	4.57	5.91	5.06	6.35	5.52	7.78	6.93	10.21	9.40
1.5	2.70	1.92	2.87	2.09	3.09	2.32	3.73	2.94	4.81	4.01
2.0	1.77	1.03	1.88	1.14	1.99	1.23	2.34	1.59	2.92	2.16
2.5	1.35	0.62	1.42	0.69	1.51	0.78	1.72	0.97	2.06	1.32
3	1.16	0.40	1.19	0.45	1.24	0.50	1.38	0.64	1.61	0.90

**Table 4:** Using covariate model, steady state ARL and SDRL results of the Bayesian CC under ME with SELF at $\lambda$ = 0.10 and cut = 5.

Shift	No error		0.1		0.2		0.5		1	
	ARL	SDRL	ARL	SDRL	ARL	SDRL	ARL	SDRL	ARL	SDRL
0.0	369.97	427.86	370.41	428.36	370.23	425.62	370.51	429.70	370.86	435.03
0.10	164.49	163.79	172.06	173.66	179.70	181.03	197.38	202.16	219.48	230.13
0.20	77.27	70.14	80.91	74.06	85.87	78.71	97.80	91.35	114.17	107.30
0.30	44.09	39.75	47.47	43.30	50.13	45.77	58.15	52.33	71.07	65.16
0.40	28.04	25.74	30.04	27.50	32.19	29.64	38.82	35.09	48.13	43.79
0.50	19.06	17.71	20.76	19.09	22.67	20.90	27.02	25.03	34.03	31.47
0.60	13.84	12.66	15.10	14.01	16.31	15.05	19.81	18.33	25.39	23.67
0.70	10.30	9.45	11.36	10.35	12.18	11.21	15.30	14.08	19.50	18.28
0.80	8.02	7.16	8.87	7.95	9.56	8.67	11.69	10.74	15.33	14.27
0.90	6.49	5.67	7.11	6.25	7.73	6.90	9.46	8.57	12.37	11.41
1.0	5.38	4.57	5.84	5.04	6.42	5.58	7.79	7.00	10.20	9.41
1.5	2.69	1.92	2.89	2.11	3.10	2.32	3.82	3.03	4.89	4.10
2.0	1.76	1.01	1.87	1.13	2.01	1.26	2.36	1.61	2.92	2.17
2.5	1.35	0.63	1.43	0.69	1.50	0.76	1.70	0.98	2.08	1.35
3	1.15	0.40	1.20	0.45	1.24	0.50	1.38	0.65	1.63	0.91

**Table 5:** Steady state ARL and SDRL in the existence of ME for covariate model using SELF, with $\lambda$ = 0.10 at cut = 20.

Shift	No error		0.1		0.2		0.5		1	
	ARL	SDRL	ARL	SDRL	ARL	SDRL	ARL	SDRL	ARL	SDRL
0.0	370.27	432.91	369.09	426.82	370.63	429.58	369.22	425.12	370.90	430.35
0.10	162.39	162.62	171.26	172.80	177.84	179.42	181.89	184.67	218.44	232.00
0.20	75.47	68.89	84.92	79.41	86.20	79.18	94.08	81.30	115.68	108.81
0.30	43.62	39.83	50.03	45.41	49.66	45.55	56.64	44.23	71.98	65.28
0.40	19.01	17.74	30.35	27.93	32.34	29.65	36.92	34.37	47.71	43.48
0.50	13.68	12.71	20.72	19.33	22.41	20.79	27.65	24.10	34.43	31.45
0.60	13.72	12.69	14.94	13.86	16.29	15.12	20.20	18.69	25.55	23.59
0.70	10.36	9.45	11.28	10.37	12.50	11.50	15.15	14.09	19.34	18.16
0.80	8.04	7.21	8.75	7.98	9.51	8.74	12.00	11.02	15.39	14.29
0.90	6.50	5.69	7.08	6.24	7.71	6.92	9.09	8.67	12.42	11.48
1.0	5.33	4.54	5.87	5.09	6.30	5.47	7.89	6.96	10.10	9.34
1.5	2.69	1.92	2.85	2.08	3.09	2.31	3.81	3.01	4.83	4.09
2.0	1.77	1.05	1.86	1.13	2.00	1.25	2.36	1.60	2.90	2.16
2.5	1.35	0.62	1.42	0.69	1.50	0.77	1.65	0.93	2.07	1.33
3	1.15	0.39	1.19	428.63	1.23	0.49	1.39	0.66	1.62	0.89

**Table 6:** For sensitivity analysis, run length outcomes of Bayesian chart applying SELF for covariate model considering ME, with different values of smoothing constant and sample sizes.

Shift	$\lambda$ = 0.25,* n* = 5			$\lambda$ = 0.25,* n* = 8			$\lambda$ = 0.30,* n* = 5			$\lambda$ = 0.30,* n* = 8		
	No ME	ME = 0.5	ME = 1	No ME	ME = 0.5	ME = 1	No ME	ME = 0.5	ME = 1	No ME	ME = 0.5	ME = 1
	ARL (SDR)	ARL (SDRL)	ARL (SDRL)	ARL (SDRL)	ARL (SDRL)	ARL (SDRL)	ARL (SDRL)	ARL (SDRL)	ARL (SDRL)	ARL (SDRL)	ARL (SDRL)	ARL (SDRL)
0.0	370.49 (351.77)	368.89 (346.62)	370.55 (360.14)	370.63 (351.81)	370.54 (350.71)	369.17 (345.15)	369.05 (342.08)	369.81 (346.43)	370.72 (349.40)	370.29 (349.32)	370.05 (347.34)	370.68 (346.66)
0.10	79.38 (54.28)	99.35 (70.42)	118.29 (87.35)	61.29 (40.38)	76.53 (51.73)	91.48 (63.62)	80.24 (52.92)	101.36 (69.91)	119.98 (86.90)	62.62 (38.78)	79.15 (50.76)	93.98 (62.42)
0.20	32.25 (20.78)	42.59 (27.23)	51.81 (33.30)	23.20 (15.26)	31.21 (20.00)	37.97 (24.24)	34.25 (20.55)	44.30 (26.88)	52.88 (32.65)	24.71 (15.04)	32.86 (19.59)	39.92 (23.89)
0.30	17.36 (11.51)	23.84 (15.35)	29.90 (18.74)	11.72 (8.01)	16.63 (10.94)	20.86 (13.60)	18.55 (11.38)	25.07 (15.12)	31.21 (18.36)	12.82 (8.05)	17.79 (10.99)	22.68 (13.44)
0.40	10.52 (7.11)	15.03 (9.89)	19.16 (12.19)	6.91 (4.71)	9.96 (6.71)	12.86 (8.51)	11.49 (7.20)	16.02 (9.76)	20.48 (12.11)	7.60 (4.84)	11.00 (6.89)	14.19 (8.62)
0.50	7.05 (4.70)	10.16 (6.70)	13.29 (8.51)	4.59 (3.01)	6.59 (4.44)	8.58 (5.69)	7.76 (4.88)	11.12 (6.90)	14.34 (8.62)	5.06 (3.20)	7.44 (4.63)	9.66 (5.91)
0.60	5.06 (3.32)	7.41 (4.84)	9.62 (6.22)	3.29 (2.08)	4.77 (3.09)	6.25 (4.08)	5.59 (3.44)	8.08 (4.98)	10.55 (6.33)	3.64 (2.22)	5.32 (3.27)	7.01 (4.25)
0.70	3.79 (2.38)	5.54 (3.54)	7.34 (4.71)	2.52 (1.49)	3.57 (2.24)	4.69 (2.98)	4.29 (2.55)	6.17 (3.67)	8.13 (4.84)	2.76 (1.60)	4.04 (2.43)	5.34 (3.18)
0.80	3.04 (1.83)	4.39 (2.71)	5.83 (3.66)	2.03 (1.14)	2.87 (1.71)	3.76 (2.32)	3.38 (1.92)	4.89 (2.83)	6.44 (3.77)	2.21 (1.21)	3.19 (1.81)	4.21 (2.42)
0.90	2.51 (1.44)	3.62 (2.14)	4.71 (2.84)	1.69 (0.86)	2.36 (1.35)	3.05 (1.77)	2.78 (1.53)	4.00 (2.24)	5.25 (2.98)	1.84 (0.93)	2.62 (1.42)	3.45 (1.90)
1.0	2.11 (1.15)	3.02 (1.70)	3.95 (2.28)	1.48 (0.69)	2.00 (1.08)	2.57 (1.44)	2.34 (1.22)	3.36 (1.80)	4.40 (2.39)	1.59 (0.75)	2.22 (1.16)	2.89 (1.52)
1.5	1.24 (0.47)	1.67 (0.74)	2.11 (0.99)	1.04 (0.20)	1.20 (0.43)	1.45 (0.62)	1.34 (0.54)	1.84 (0.79)	2.32 (1.03)	1.06 (0.24)	1.29 (0.50)	1.62 (0.68)
2.0	1.03 (0.17)	1.20 (0.42)	1.47 (0.59)	1.00 (0.02)	1.02 (0.14)	1.10 (0.31)	1.05 (0.22)	1.30 (0.48)	1.61 (0.62)	1.00 (0.04)	1.03 (0.19)	1.17 (0.38)
2.5	1.00 (0.03)	1.03 (0.19)	1.16 (0.37)	1.00 (0.00)	1.00 (0.02)	1.01 (0.10)	1.00 (0.05)	1.07 (0.25)	1.24 (0.44)	1.00 (0.00)	1.00 (0.04)	1.02 (0.05)
3	1.00 (0.00)	1.00 (0.06)	1.03 (0.19)	1.00 (0.00)	1.00 (0.00)	1.00 (0.02)	1.00 (0.00)	1.00 (0.09)	1.07 (0.25)	1.00 (0.00)	1.00 (0.00)	1.00 (0.03)

**Table 7:** The run length profiles for Bayesian chart under ME under SELF for multiple measurement method, with $\lambda$ = 0.10.

Shift	No Error		0.1		0.2		0.5		1	
	ARL	SDRL	ARL	SDRL	ARL	SDRL	ARL	SDRL	ARL	SDRL
0.0	369.64	424.64	370.26	431.09	372.68	428.45	373.19	431.82	372.18	425.61
0.10	165.37	165.69	167.96	166.11	171.96	172.06	172.73	174.70	177.30	180.99
0.20	75.82	69.27	77.63	71.06	79.08	72.04	81.31	74.77	84.86	78.53
0.30	43.64	39.96	44.66	40.76	45.83	41.74	47.39	42.89	50.04	45.44
0.40	28.04	25.68	28.07	25.90	29.15	26.63	30.25	27.84	32.30	29.79
0.50	19.20	17.86	19.37	17.90	19.92	18.50	20.98	19.42	22.22	20.52
0.60	13.79	12.78	13.97	12.83	14.37	13.17	14.98	13.90	16.26	15.01
0.70	10.33	9.43	10.59	9.68	10.80	9.90	11.30	10.33	12.18	11.25
0.80	8.04	7.22	8.26	7.39	8.36	7.56	8.87	7.97	9.72	8.81
0.90	6.56	5.70	6.62	5.75	6.77	5.93	7.11	6.27	7.66	6.86
1.0	5.37	4.58	5.45	4.61	5.54	4.73	5.88	5.09	6.29	5.47
1.5	2.69	1.91	2.73	1.98	2.76	1.99	2.91	2.13	3.12	2.34
2.0	1.76	1.03	2.73	1.98	1.82	1.08	1.89	1.15	1.99	1.25
2.5	1.35	0.63	1.38	0.64	1.39	0.66	1.43	0.70	1.51	0.79
3	1.15	0.40	1.16	0.40	1.17	0.42	1.20	0.45	1.24	0.50

**Table 8:** The run length profiles for Bayesian AEWMA CC in the presence of ME using P distribution under LLF for multiple measurement method, with $\lambda$ = 0.10.

Shift	No Error		0.1		0.2		0.5		1	
	ARL	SDRL	ARL	SDRL	ARL	SDRL	ARL	SDRL	ARL	SDRL
0.0	370.83	432.54	369.61	430.50	371.36	433.89	372.87	425.86	370.41	432.59
0.10	164.63	163.35	165.60	163.86	167.76	165.78	170.86	172.62	181.11	180.26
0.20	76.89	69.61	76.82	70.60	77.84	72.19	81.17	74.10	86.94	79.57
0.30	43.21	39.75	44.47	40.19	45.10	41.45	47.12	42.50	49.59	45.07
0.40	27.91	25.54	28.51	26.25	29.50	26.84	30.48	27.93	32.15	29.46
0.50	19.23	17.75	19.55	18.07	20.10	18.45	20.83	19.47	22.40	20.57
0.60	13.86	12.81	14.08	13.01	14.37	13.36	14.94	13.86	16.30	14.94
0.70	10.39	9.42	10.49	9.61	10.91	9.99	11.29	10.49	12.41	11.33
0.80	8.03	7.24	8.22	7.40	8.43	7.60	8.86	7.97	9.62	8.71
0.90	6.46	5.66	6.67	5.84	6.85	6.00	7.14	6.28	7.65	6.87
1.0	5.41	4.58	5.48	4.68	5.61	4.77	5.80	5.05	6.34	5.50
1.5	2.67	1.91	2.73	1.97	2.79	2.02	2.90	2.13	3.11	2.36
2.0	1.77	1.02	1.79	1.06	1.83	1.09	1.88	1.15	2.00	1.25
2.5	1.35	0.62	1.38	0.64	1.40	0.66	1.43	0.70	1.50	0.76
3	1.16	0.40	1.16	0.41	1.18	0.43	1.20	0.45	1.24	0.51

**Table 9:** The run length profiles of AEWMA chart applying ME under LLF for multiple measurement method, with $\lambda$ = 0.10.

Shift	No Error		0.1		0.2		0.5		1	
	ARL	SDRL	ARL	SDRL	ARL	SDRL	ARL	SDRL	ARL	SDRL
0.0	369.85	428.77	372.64	429.22	371.39	426.37	370.89	422.74	370.63	421.58
0.10	167.43	168.38	169.26	167.58	165.73	165.61	172.34	172.89	180.41	182.52
0.20	76.05	69.72	76.58	70.03	78.20	71.30	80.79	74.42	85.98	78.30
0.30	43.51	39.68	44.34	40.37	44.73	40.87	46.92	43.06	50.29	45.92
0.40	28.16	25.83	28.39	26.20	28.57	26.48	29.82	27.56	32.30	29.78
0.50	18.96	17.63	19.44	17.96	19.68	18.21	20.79	19.36	22.27	20.61
0.60	13.73	12.52	14.08	13.06	14.37	13.17	14.94	13.88	16.14	15.06
0.70	10.40	9.50	10.69	9.74	10.97	9.98	11.32	10.36	12.44	11.29
0.80	8.22	7.35	8.14	7.36	8.45	7.62	8.85	8.05	9.68	8.81
0.90	6.45	5.62	6.68	5.81	6.77	5.92	7.09	6.32	7.70	6.87
1.0	5.35	4.53	5.46	4.63	5.57	4.80	5.80	5.03	6.32	5.55
1.5	2.66	1.91	2.75	1.98	2.76	2.01	2.90	2.11	3.13	2.35
2.0	1.77	1.03	1.78	1.03	1.79	1.01	1.88	1.13	2.02	1.27
2.5	1.35	0.62	1.38	0.65	1.38	0.64	1.42	0.69	1.50	0.76
3	1.16	0.40	1.17	0.41	1.18	0.42	1.19	0.44	1.25	0.51

**Table 10:** The run length profiles for Bayesian CC with ME applying SELF for linearly related variance, with $\lambda$ = 0.10.

Shift	No error		2		3		4		5	
	ARL	SDRL	ARL	SDRL	ARL	SDRL	ARL	SDRL	ARL	SDRL
0.0	369.78	427.66	367.39	431.35	375.75	438.05	373.09	442.48	372.16	437.93
0.10	320.51	374.19	346.96	401.29	354.79	413.62	357.98	415.47	360.09	422.39
0.20	244.55	265.23	289.21	324.90	312.98	357.39	280.06	313.35	333.08	384.08
0.30	179.79	187.33	235.91	250.54	263.31	289.65	239.99	258.27	295.65	328.56
0.40	135.48	134.53	186.45	192.93	219.61	234.65	204.54	215.07	258.07	282.30
0.50	107.09	102.97	152.44	151.38	181.76	187.95	206.46	217.29	221.39	236.61
0.60	85.90	81.29	128.04	124.77	155.23	155.18	176.38	179.89	194.14	200.46
0.70	72.38	67.90	105.61	102.21	133.25	130.71	153.31	152.67	171.42	174.49
0.80	60.20	55.97	91.72	87.15	115.00	110.23	134.42	132.59	149.35	149.47
0.90	50.55	47.49	79.15	74.86	100.91	96.21	117.51	113.24	132.24	130.72
1.0	43.80	41.41	69.94	65.13	89.45	84.64	104.64	99.31	119.14	114.87
1.5	22.57	21.84	38.37	36.83	52.91	49.63	63.44	59.42	73.32	69.02
2.0	13.62	13.09	24.13	22.98	33.40	31.77	41.18	39.32	49.58	46.84
2.5	8.95	8.50	16.14	15.83	23.40	22.64	29.11	28.07	35.25	33.58
3	6.30	5.87	11.42	11.19	16.68	16.37	21.43	20.82	26.36	25.25

**Table 11:** The run length profiles for Bayesian-AEWMA chart under ME with LLF for linearly related variance, with $\lambda$ = 0.10.

Shift	No error		2		3		4		5	
	ARL	SDRL	ARL	SDRL	ARL	SDRL	ARL	SDRL	ARL	SDRL
0.0	370.01	437.93	363.50	431.62	369.01	436.59	369.72	427.55	369.77	433.64
0.10	332.07	378.18	337.63	391.13	354.03	417.51	356.59	422.93	359.36	418.72
0.20	247.51	269.00	284.00	320.93	311.71	349.17	322.73	371.80	329.39	381.37
0.30	183.55	188.84	228.01	244.46	263.05	286.88	278.84	313.85	288.20	322.28
0.40	140.19	135.57	181.38	189.27	214.32	229.39	241.28	264.70	256.25	279.95
0.50	108.55	103.97	151.27	151.88	182.49	188.78	201.39	211.33	224.23	239.55
0.60	87.68	81.70	124.42	122.41	154.66	155.12	175.35	178.33	194.93	203.03
0.70	73.45	68.35	104.66	101.49	134.55	133.21	154.28	153.68	165.71	168.81
0.80	60.84	57.08	91.15	87.65	114.60	111.29	133.45	132.75	149.21	150.26
0.90	51.31	47.68	79.02	74.40	102.32	96.81	118.07	115.68	131.18	129.25
1.0	44.19	41.57	68.85	64.46	88.66	83.50	104.64	99.47	117.77	114.11
1.5	22.69	21.92	38.69	36.79	51.29	48.19	63.24	59.34	73.20	69.94
2.0	13.74	13.06	24.40	23.47	33.47	32.24	41.97	39.64	49.61	46.63
2.5	8.87	8.48	16.01	15.58	22.95	22.28	29.03	28.04	35.40	33.51
3	6.29	5.83	11.40	11.12	16.66	16.18	21.51	20.85	25.90	69.94

**Table 12:** The run length profiles for Bayesian CC applying ME using PP distribution under LLF for linearly related variance, with $\lambda$ = 0.10.

Shift	No error		2		3		4		5	
	ARL	SDRL	ARL	SDRL	ARL	SDRL	ARL	SDRL	ARL	SDRL
0.0	369.62	428.47	365.38	424.63	371.35	431.38	369.27	429.99	372.38	442.00
0.10	314.86	357.08	340.25	393.47	355.43	414.54	356.60	415.97	361.89	421.06
0.20	242.97	264.33	284.35	320.12	311.80	348.48	**322.49**	375.79	335.07	382.75
0.30	178.92	182.169	232.09	250.56	262.85	291.29	281.74	316.83	291.36	327.31
0.40	134.90	133.98	181.58	189.38	218.13	232.38	235.91	257.55	259.62	284.85
0.50	107.85	102.18	150.03	151.47	181.16	190.09	204.80	215.81	219.34	236.31
0.60	85.51	81.00	124.31	120.56	152.99	153.78	177.81	182.65	194.16	201.77
0.70	71.72	67.56	105.59	101.57	131.21	128.85	152.95	153.22	169.73	172.02
0.80	59.56	55.86	91.36	86.40	114.19	110.11	133.92	131.85	148.21	147.70
0.90	50.96	47.54	78.95	74.66	102.11	96.57	117.55	114.45	131.36	129.16
1.0	43.06	40.62	69.60	66.31	89.53	84.67	104.45	100.85	119.02	115.55
1.5	22.56	21.77	38.34	36.61	52.09	49.20	63.72	59.68	73.01	69.41
2.0	13.36	9.94	23.70	23.19	33.46	32.22	41.70	39.75	49.49	46.94
2.5	8.79	8.39	16.26	15.77	23.44	22.57	29.07	28.05	34.87	33.78
3	6.30	5.85	11.58	11.17	16.58	16.19	21.47	20.70	26.20	25.21

This section of the study delves deeply into the content of these tables, embarking on a comprehensive exploration that reveals significant insights stemming from the utilization of the AEWMA CC method within the Bayesian framework. Notably, the versatility of the approach is underscored by its adaptation to diverse LFs, thereby enriching the overall understanding of the underlying phenomena. Tables 1, 2, 3, 4, 5, 6, 7, 8 and 9 indicate that as the shift magnitude increases from 0.10 to 0.20 and continues up to 3, both the ARL and SDRL values decrease. This decline suggests that even minor, moderate, and substantial changes in the process parameter are detected at an earlier stage. This conclusion is supported by the reduced ARL values for each shift compared to their previous values. This trend culminates in an ARL approaching unity at shift 3. These consistent patterns persist across all tables, irrespective of the presence of errors (ranging from none to magnitudes of 0.1, 0.2, 0.5, or 1). These observations underscore a fundamental trait of AEWMA CCs.

**Figure 1:**
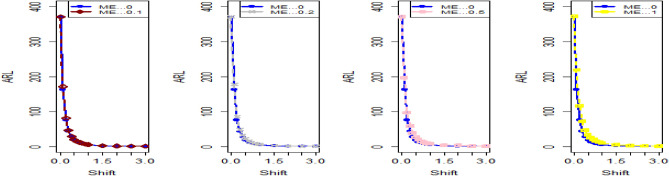
Under covariate model, the ARL plots for the suggested CC using SELF consider values of $\frac{{\delta_{m}^{2} }}{{\delta^{2} }}$.

**Figure 2:**
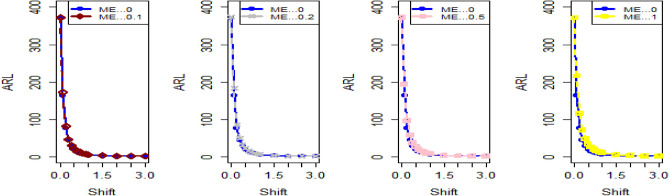
Utilizing covariate model, ARL plots for the suggested CC using LLF with various values of $\frac{{\delta_{m}^{2} }}{{\delta^{2} }}$.

**Figure 3:**
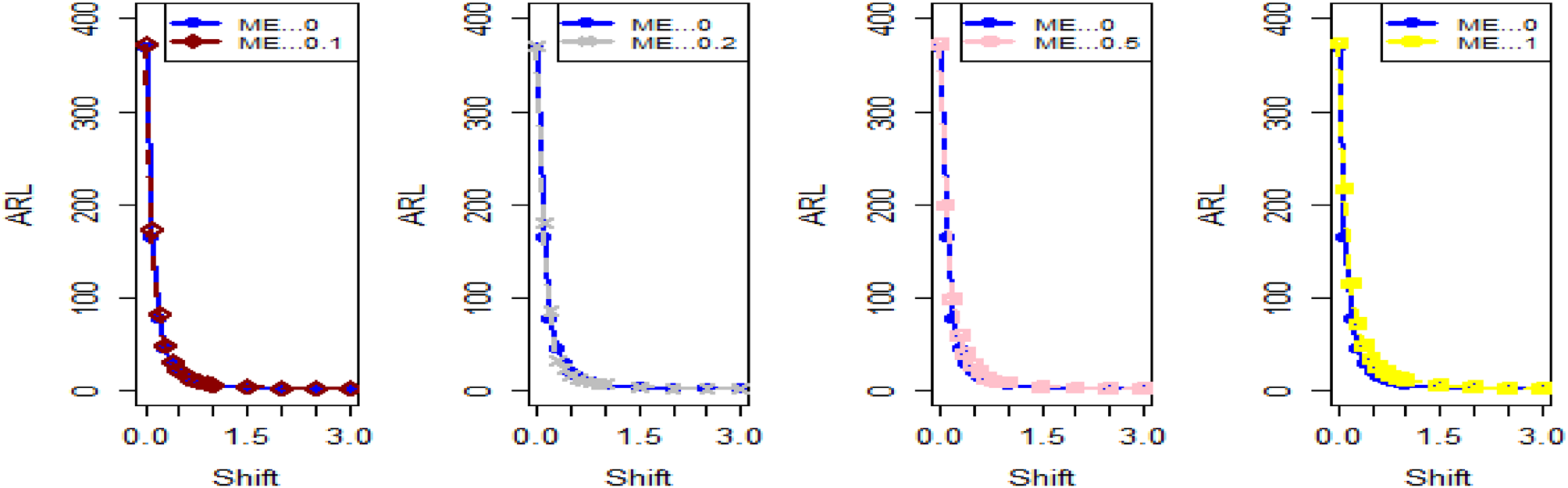
Using covariate model, ARL plots for the suggested CC for P and PP distribution using various values of $\frac{{\delta_{m}^{2} }}{{\delta^{2} }}$.

Shifting our focus to the impact of ME on CC efficiency, a consistent pattern emerges from the analysis of the tables. As the magnitude of error escalates from zero to 0.2 and further to a unit value, a corresponding increase is observed in ARLs. This increase leads to delays in detecting process shifts when employing methods to address ME. Consequently, it becomes evident that ME exerts a detrimental effect on the effectiveness of Bayesian AEWMA CCs in identifying process shifts within the context of industrial production. In Table 1, we are able to closely scrutinize the outcomes pertaining to the ARL outcomes of the Bayesian AEWMA CC method as proposed. This table provides a comprehensive display of these outcomes based on the recommended designs utilizing SELF for the covariate model, while considering specific parameter values, namely A = 0 and B = 1. Moreover, the table offers a presentation of the results across various scenarios characterized by different levels of ME. These scenarios include cases with no error, an ME value of 0.5, and an ME value of 1. Upon closer examination, a discernible pattern becomes apparent: the run length values exhibit an upward trend with an increase in the value of ME. In simpler terms, as the magnitude of ME rises, the associated run length values also increase. This pattern suggests that higher levels of ME have an influence on the detection efficiency of the Bayesian CC, potentially leading to delays in monitoring the shifts in the process mean. For example under SELF, at $\frac{{\delta_{m}^{2} }}{{\delta^{2} }}$ = 0.0, 0.1, 0.2, 0.5 and 1 with $\lambda = 0.10$ and shift $\sigma = 0.30$, the ARL values are 43.86, 46.95, 49.91, 58.50 and 71.93, and for shift $\sigma = 0.80$, ARL values are 8.05, 8.81, 9.55, 11.76, and 15.32. The same pattern is observed in the Table 2 for P distribution and Table 3 for PP distributions under LLF. *i.e.,* From Table 3 at $\frac{{\delta_{m}^{2} }}{{\delta^{2} }}$ = 0.0, 0.1, 0.2, 0.5 and 1 using $\lambda = 0.10$ with $\sigma = 0.30$ the run length values are 43.99, 47.30, 52.34, 58.18, and 70.57. Also, we show the same efficiency in the Figs. 1, 2 and 3, which presents the ARL plots using covariate method for the suggested design.

**Figure 4:**
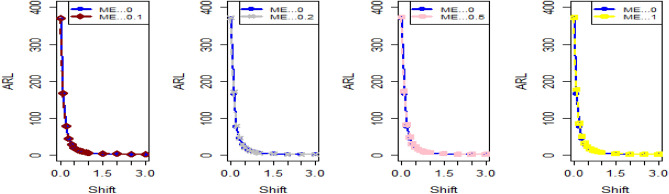
Using multiple measurements, the ARL plots for the proposed CC using SELF consider values of $\frac{{\delta_{m}^{2} }}{{\delta^{2} }}$.

**Figure 5:**
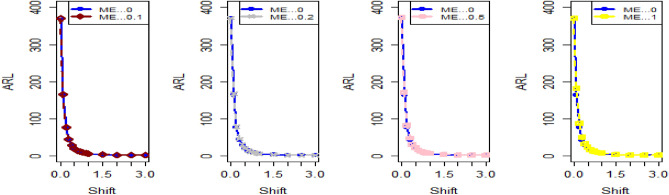
Utilizing multiple measurements, ARL plots for the recommended CC using LLF with different values of $\frac{{\delta_{m}^{2} }}{{\delta^{2} }}$.

**Figure 6:**
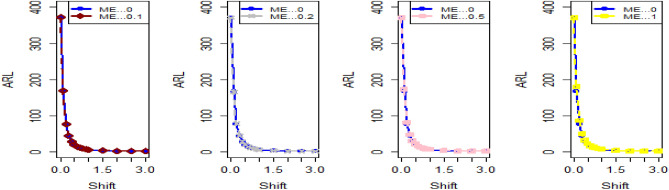
Under LLF, ARL plots for the suggested CC for P and PP distribution using distinct values of $\frac{{\delta_{m}^{2} }}{{\delta^{2} }}$.

**Figure 7:**
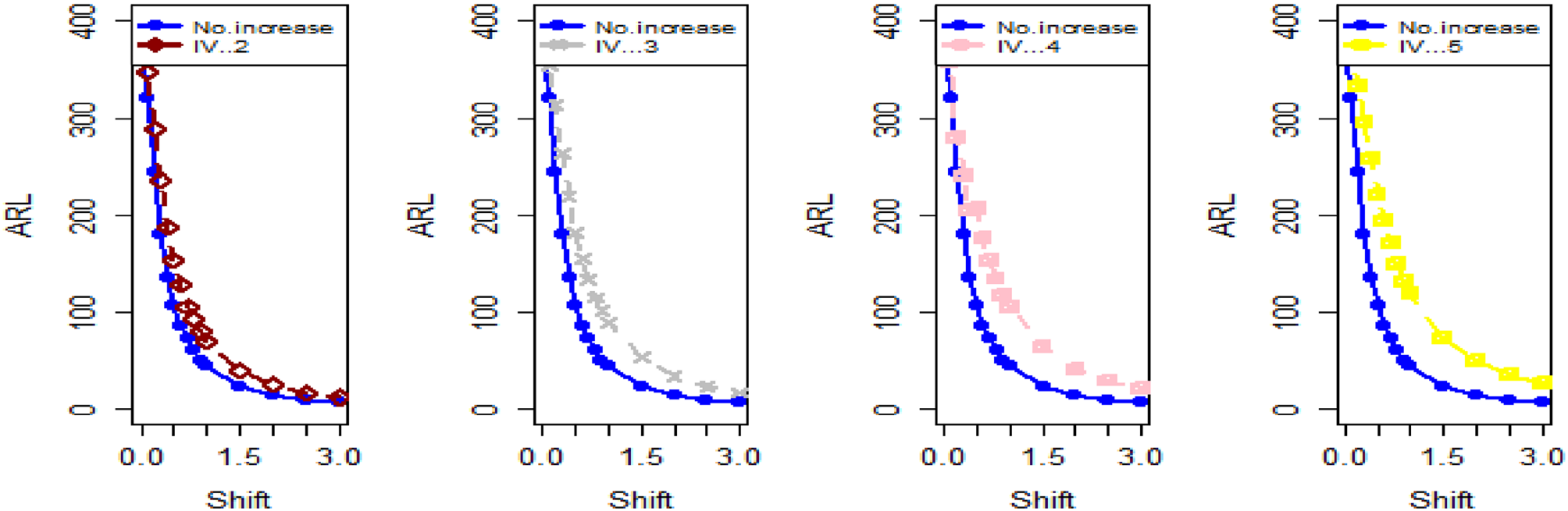
Based on the linearly increasing method, the ARL plots for the suggested CC applying SELF consider values of *D*.

**Figure 8:**
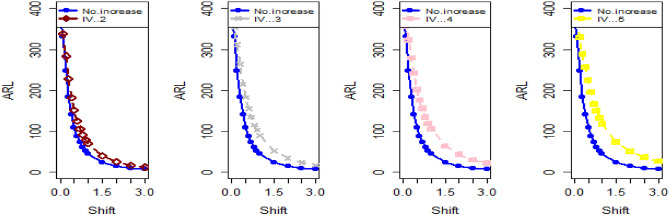
Using LLF, ARL plots for the suggested CC applying linearly increasing variance method with values of *D*.

**Figure 9:**
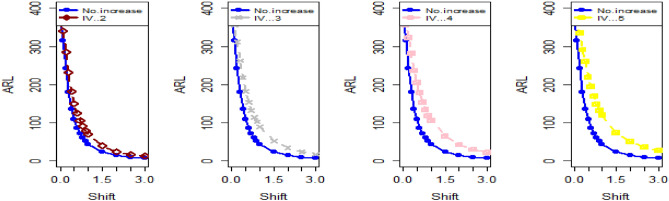
Using LLF, ARL plots for the suggested CC applying linearly increasing variance method with values of *D* for P and PP distribution.

The data in Tables 7, 8 and 9 illustrates the significant impact of conducting multiple measurements on the same sample in mitigating the ME effect. Comparing the run length outcomes between Tables 4, 5,6 and Tables 1, 2, 3 demonstrates that the utilization of multiple measurements effectively minimizes the ME effect. This enhancement in chart efficiency enables the early detection of process shifts. As an illustration, under SELF Table 7, when $\frac{{\delta_{m}^{2} }}{{\delta^{2} }}$ = 0.0, 0.1, 0.2, 0.5, and 1 are applied in conjunction with $\lambda = 0.10$ and shift $\sigma = 0.30$, the corresponding ARL values are 43.64, 44.66, 45.83, 47.39, and 50.04. Notably, these values are substantially lower than those detailed in Table 1, as discussed previously. The Figs. 4, 5 and 6 show the ARL plots for the multiple measurements method and indicate the same efficiency. Table 10, 11 and 12 presents the ARL and SDRL values for the Bayesian AEWMA CC applied to a scenario involving linearly increasing variance. The experiments entail varying the parameter D across different LFs. Notably, In Table 10, the ARL values at δ = 0.3 are observed to be 179.79 and 263.31 for D = 0 and 3, respectively, within the SELF condition. This trend suggests a decrease in the efficiency of the Bayesian EWMA control chart as the value of D increases. Additionally, Figs. 7, 8 and 9 also indicate an increase in ARL values with an increase in the value of D. Table 6 provides insights into the sensitivity of the suggested CC when confronted with ME while examining various smoothing constant values and sample sizes. The data clearly illustrates those higher smoothing constant values correspond to increased ARL values, indicating the CC's inefficiency in handling ME. Moreover, an increase in the sample size leads to a noticeable decline in the resulting ARL values across different shift values.

Based on the preceding dialogue, we can formulate the key discoveries at this juncture.

- The efficacy of AEWMA CCs in detecting minor to moderate process shifts is apparent from the ARL and SDRL values presented in all nine tables for the proposed strategy.
- The previous discussion also addressed the negative effect of ME on the effectiveness of the proposed CCs.
- The decrease in error effect resulting from multiple measurements is apparent from the ARL values in Tables 7, 8 and 9 and the earlier discussions about these tables. This confirms the effectiveness of utilizing multiple measures to alleviate the error effect in our recommended CCs.
- By analyzing P and PP distributions using the Bayesian framework, it becomes clear that the newly introduced Bayesian AEWMA CC, when implemented with multiple measurements and in the presence of ME, shows reduced vulnerability to ME compared to the other two methods. These observations stem from the utilization of informative priors and the integration of both LFs.

## Real life data applications

This article illustrates the practical implementation of the recommended Bayesian AEWMA CC in the context of ME. The specific dataset utilized for this purpose is drawn from Montgomery^27^ and focuses on the hard-bake process in semiconductor production. Consisting of a total of 45 samples, with each sample comprising 5 wafers, the dataset amounts to 225 data points in total. These data points represent measurements of flow width and are expressed in microns. The time interval between each sample is uniformly set at one hour. The initial 35 samples, making up 175 observations, are identified as representative of the controlled process and are designated as the phase-I dataset. In contrast, the remaining 10 samples, totaling 50 observations, are categorized as indicative of the out-of-control process and are labeled as the phase-II dataset. The Phase I dataset can be employed to estimate the parameters necessary for determining control limits. Typically, these control limits are derived from the stable data collected during Phase I. Subsequently, the same dataset is utilized to create the chart. If all the data points fall within the established control limits, these limits can then be carried over to Phase II. In cases where data points extend beyond the control limits, out-of-control points are identified and removed, after which new control limits are constructed for Phase II implementation.

**Figure 10:**
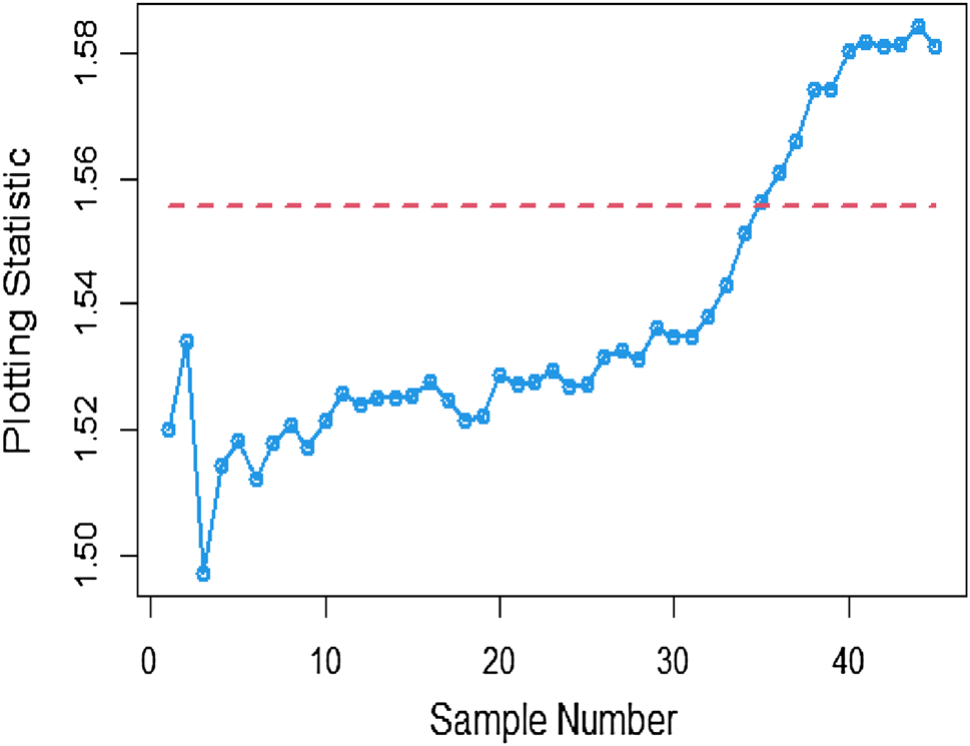
Applying covariate model, Bayesian chart with SELF at $\frac{{\delta_{m}^{2} }}{{\delta^{2} }} = 0$.

**Figure 11:**
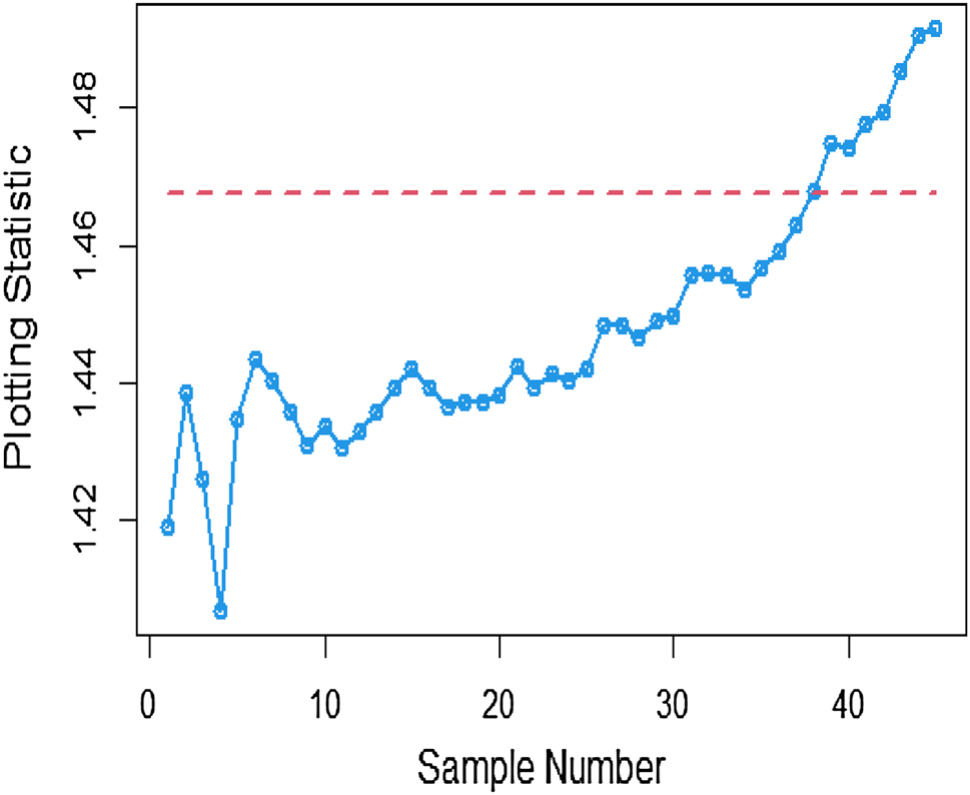
Utilizing covariate model, Bayesian CC by utilizing SELF at $\frac{{\delta_{m}^{2} }}{{\delta^{2} }} = 0.3.$

**Figure 12:**
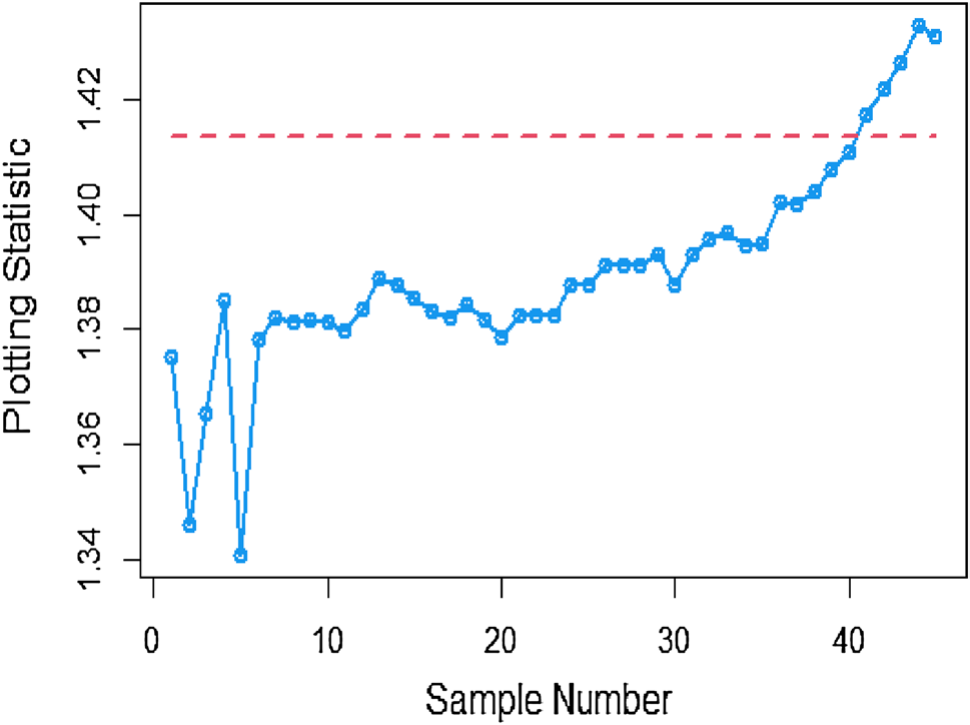
Suggested CC for covariate model using SELF at $\frac{{\delta_{m}^{2} }}{{\delta^{2} }} = 0.5.$

To apply the suggested Bayesian AEWMA Chart using covariate models with SELF, we examine different error ratio $\frac{{\delta_{m}^{2} }}{{\delta^{2} }}$ values are: 0.0, 0.5, and 1. The results of recommended chart applying covariate model with SELF are displayed in Figs. 10, 11, and 12. The respective error ratio $\frac{{\delta_{m}^{2} }}{{\delta^{2} }}$ values of 0.0, 0.3, and 0.5 are considered. After careful examination, it becomes clear that the process strays from its controlled state in the 35th, 39th, and 41st samples.

**Figure 13:**
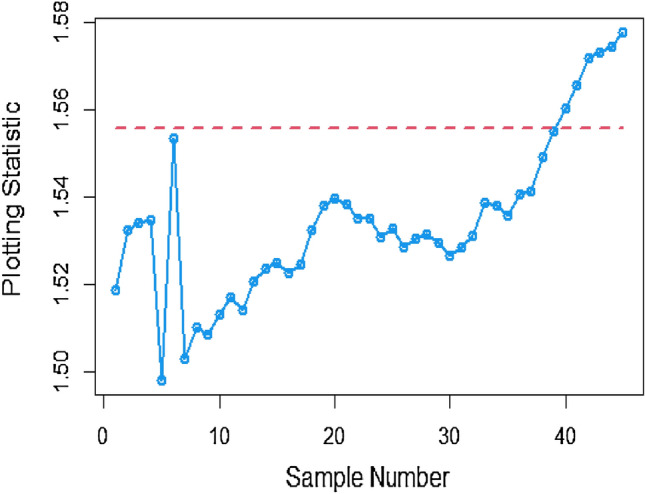
Utilizing SELF, AEWMA CC for multiple measurements method at $\frac{{\delta_{m}^{2} }}{{\delta^{2} }} = 0$.

**Figure 14:**
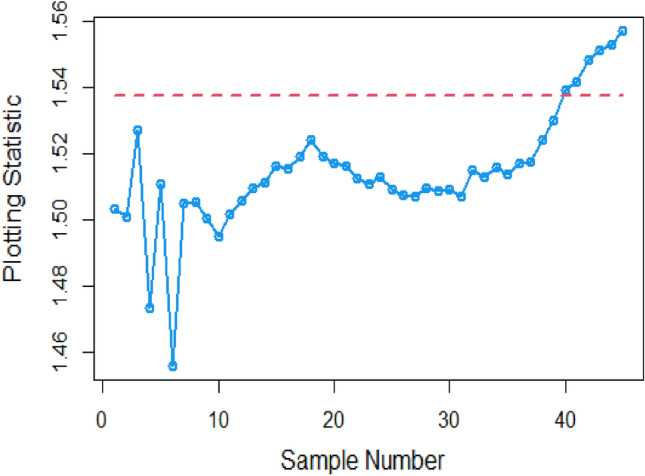
AEWMA CC with multiple measurement method under SELF for $\frac{{\delta_{m}^{2} }}{{\delta^{2} }} = 0.3.$

**Figure 15:**
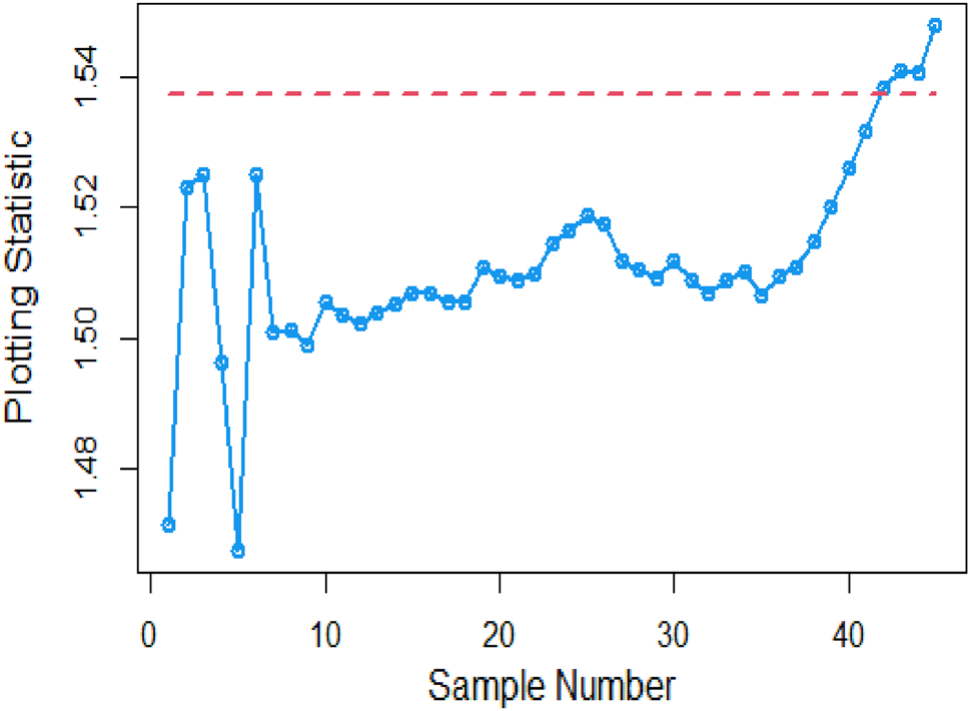
Bayesian CC by using SELF for multiple measurement method at $\frac{{\delta_{m}^{2} }}{{\delta^{2} }} = 0.5.$

**Figure 16:**
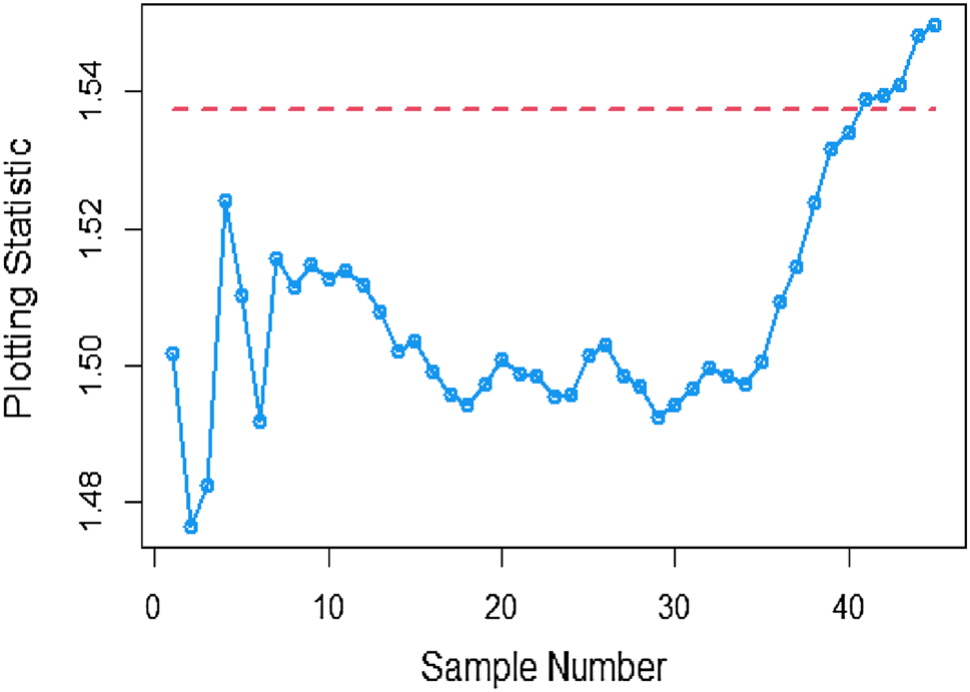
Bayesian CC using linearly increasing variance with SELF for *D* = 1.

**Figure 17:**
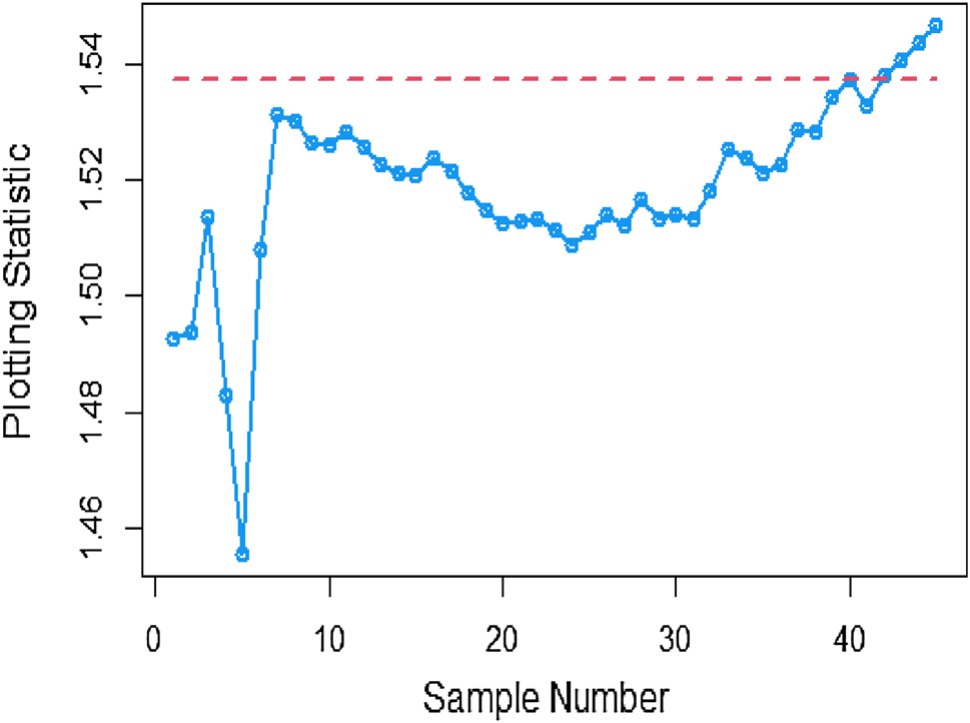
Bayesian AEWMA CC applying linearly increasing variance under SELF for *D* = 3.

**Figure 18:**
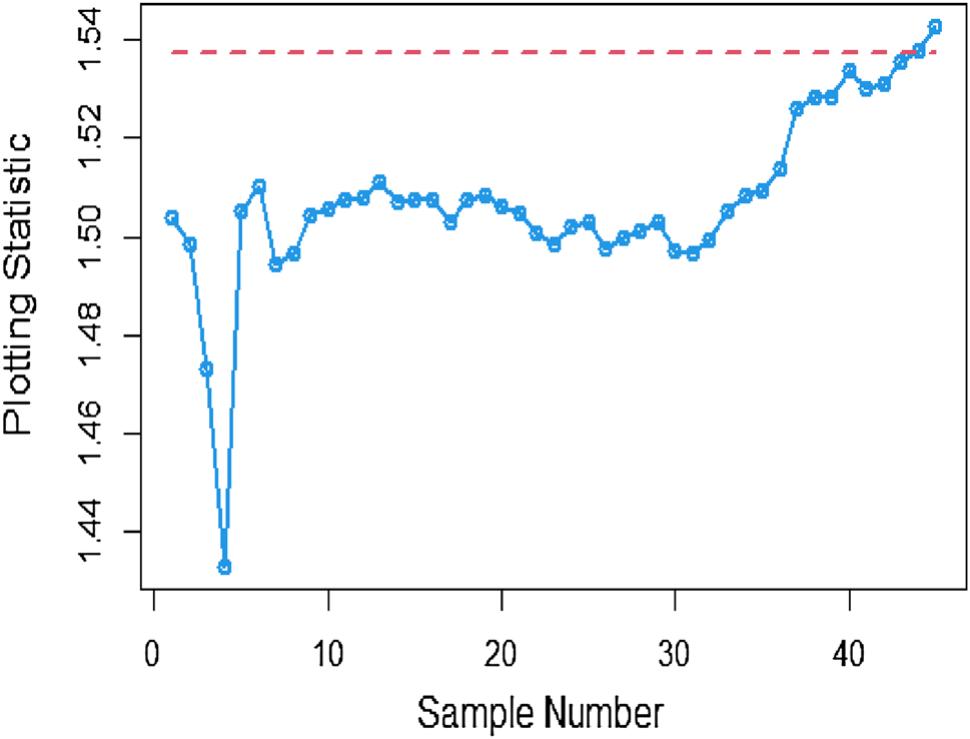
Suggested CC applying linearly increasing variance under SELF for *D* = 5.

Figures 13, 14, and 15 showcase the execution of the suggested CC utilizing Multiple measurement method incorporating the SELF. The charts present results for various error ratios: 0.0, 0.3, and 0.5. Upon a detailed examination of these charts, it is revealed that the process shows signs of being out of control in the 36th, 38th, and 40th samples. Additionally, Figs. 16, 17, and 18 provide a visual representation of the performance of the suggested CC utilizing the SELF. These figures clearly demonstrate that the process exhibits indications of being out of control in the 37th, 40th, and 42nd samples in the same context. This highlights that the MRSS scheme is the most effective compared to the other two schemes in terms of the efficacy of control charts for detecting process shifts during industrial production.

## Conclusion

This study focuses on examining how ME affects the AEWMA CC using Bayesian techniques, specifically incorporating various LFs such as SELF and LLF. To assess the performance of the proposed CC with ME, two metrics are employed, ARL and SDRL. The results of ARL offer valuable information on the simulation results of Bayesian CCs employing three different strategies to address ME: the covariate approach, multiple measurements, and the linearly increasing variance. The results indicate that the suggested Bayesian AEWMA CC, when implemented with a multiple measurement design, demonstrates improved effectiveness compared to alternative methods in scenarios involving ME. As a result, the research recommends adopting suggested chart with multiple measurements for robust monitoring of shifts in process mean, particularly when ME is a contributing factor.

### Limitations of the study and future recommendations

Implementing Bayesian CCs in the presence of ME often encounters computational challenges, particularly with large sample sizes, requiring resource-intensive procedures. Moreover, the reliance on carefully selected prior distributions for the process mean and variance can introduce subjectivity and potential bias within the Bayesian approach. Furthermore, the assumption of data exchangeability in the Bayesian-AEWMA CC with ME can yield unreliable results, demanding a thorough evaluation of data exchangeability prior to implementation. The proposed Bayesian CCs, when applied under ME, offer a versatile approach that can be extended to other memory-type control charts, allowing the adaptation of the methodology to accommodate diverse probability distributions beyond the normal distribution, such as Poisson or binomial distributions. Nevertheless, these extensions necessitate modifications to the likelihood function during the Bayesian updating procedure. By incorporating these adjustments, the methodology becomes adaptable to a broader range of data distributions, enhancing its applicability and potential impact across various industries. This adaptability is particularly valuable in sectors like healthcare, finance, and manufacturing, where diverse data distributions are encountered, enabling more effective and efficient quality control processes.

### Supplementary Information

Supplementary Information.

## Data Availability

If there is a reasonable request, the corresponding author is able to provide the datasets that were used and/or scrutinized during the course of the present study.
