# Supplementary material for: Monitoring the process mean under the Bayesian approach with application to hard bake process
Source: Sci Rep. 2023 Nov 25;13:20723. doi: 10.1038/s41598-023-48206-1 (PMC10676415; doi:10.1038/s41598-023-48206-1)
Supplement: Supplementary file 1 — Supplementary Information. [file 41598_2023_48206_MOESM1_ESM.docx]

Appendix A

Appendix A.1

Bayes estimator for the Bayesian AEWMA CC under covariate model with ME under LLF for P distribution.

|  | (A) |
| --- | --- |

Appendix A.2

Bayes estimator for the Bayesian AEWMA CC under covariate model with ME under LLF for PP distribution.

|  | (A) |
| --- | --- |

Appendix A.3

Bayes estimator for the Bayesian AEWMA CC under multiple measurements method with ME under LLF for P distribution.

|  |  |
| --- | --- |

Appendix A.4

Bayes estimator for the Bayesian AEWMA CC under multiple measurements method with ME under LLF for PP distribution.

|  |  |
| --- | --- |

**Appendix A**

#ARL using posterior and predictive posterior dis under SELF

#correct version of ARL using posterior and predictive posterior dis under SELF

library(MASS)

Mx=c(); Zp=c(); ucl=c(); lcl=c(); rl=c();

muy=0; mux=0; sigyy=1; sigm=0.0

n=1;

Wp=c();sci=c();

ld=0.10; B=1;A=0; h = 0.0681; delta=0.0; sigmp=c(); sigmt=c(); a = 7;

# Baysian Part

m_pr=0;sd_pr=1

m_po=A+B*muy

vr=(B^2*sigyy+sigm)

# Under Self

NU=(n*m_po*(sd_pr^2)+(vr)*m_pr)

DE=vr+n*sd_pr^2

muM = muW = NU/DE

sig=1

sigma=(n*vr*(sd_pr)^4)/DE^2

shift=A+B*(muy+delta*sqrt(sigyy));

for(j in 1:20000)

{

for(i in 1:1000000)

{

y=rnorm(n,shift,sqrt(vr))

NUs=(n*mean(y)*(sd_pr)+(vr)*m_pr)

DEs=vr+n*sd_pr

Mx[i]=NUs/DEs

if(i==1)

{Zp[i]=ld*Mx[i]+ (1-ld)*muM;}

else{Zp[i]=ld*Mx[i]+(1-ld)*Zp[i-1];}

sigmp[i]=(Zp[i]/(1-(1-ld)^i))

sigmt[i]=abs(sigmp[i])

if(sigmt[i] > 0 && sigmt[i] <= 1)

{

sci = 1/(a*(1+(sigmt[i])^(-2)))

}

else if(sigmt[i] > 1 && sigmt[i] <= 2.7)

{

sci = 1/(a*(1+(sigmt[i])^(-1)))

}

else

{

sci=1.00

}

if(i==1)

{Wp[i]=sci*Mx[i]+ (1-sci)*muW;}

else{Wp[i]=sci*Mx[i]+(1-sci)*Wp[i-1];}

if(abs(Wp[i])>h)

{rl[j]=i;break;}

else{rl[j]=0;}

}

}

mean(rl)

sd(rl)

*********************************************************************************

**R Codes for Steady-State ARL**

**#ARL using posterior and predictive posterior dis under SELF**

**#correct version of ARL using posterior and predictive posterior dis under SELF**

library(MASS)

Mx=c(); Zp=c(); ucl=c(); lcl=c(); rl=c();

muy=0; mux=0; sigyy=1; sigm=0.0

n=1;

Wp=c();sci=c();

ld=0.10; B=1;A=0; h = 0.0681; delta=0.0; sigmp=c(); sigmt=c(); a = 7;

# Baysian Part

m_pr=0;sd_pr=1

m_po=A+B*muy

vr=(B^2*sigyy+sigm)

# Under Self

NU=(n*m_po*(sd_pr^2)+(vr)*m_pr)

DE=vr+n*sd_pr^2

muM = muW = NU/DE

sig=1

sigma=(n*vr*(sd_pr)^4)/DE^2

shift=A+B*(muy+delta*sqrt(sigyy));

for(j in 1:20000)

{

for(i in 1:1000000)

{

y=rnorm(n,shift,sqrt(vr))

NUs=(n*mean(y)*(sd_pr)+(vr)*m_pr)

DEs=vr+n*sd_pr

Mx[i]=NUs/DEs

if(i==1)

{Zp[i]=ld*Mx[i]+ (1-ld)*muM;}

else{Zp[i]=ld*Mx[i]+(1-ld)*Zp[i-1];}

sigmp[i]=(Zp[i]/(1-(1-ld)^i))

sigmt[i]=abs(sigmp[i])

if(sigmt[i] > 0 && sigmt[i] <= 1)

{

sci = 1/(a*(1+(sigmt[i])^(-2)))

}

else if(sigmt[i] > 1 && sigmt[i] <= 2.7)

{

sci = 1/(a*(1+(sigmt[i])^(-1)))

}

else

{

sci=1.00

}

if(i==1)

{Wp[i]=sci*Mx[i]+ (1-sci)*muW;}

else{Wp[i]=sci*Mx[i]+(1-sci)*Wp[i-1];}

if(abs(Wp[i])>h & j>=20)

{rl[j]=i;break;}

else{rl[j]=0;}

}

}

mean(rl)

sd(rl)
